# Prepared on Paper, Unprepared in Practice: The Transition From Medical Student to Resident Doctor

**DOI:** 10.7759/cureus.111754

**Published:** 2026-06-29

**Authors:** Vedant Lal

**Affiliations:** 1 Urology, Russells Hall Hospital, Birmingham, GBR

**Keywords:** medical education, medical student preparedness, medical students, resident doctors, transition to clinical practice

## Abstract

The transition from medical student to resident doctor is a difficult phase in medical training. Undergraduate medical education often provides a strong foundation in clinical knowledge. However, many newly qualified doctors report difficulty applying this in clinical environments. A national UK study analysing qualitative transition narratives found that 30.3% of accounts were explicitly classified as 'unprepared,' outnumbering those classified as 'prepared' (23.2%). Furthermore, a major UK multi-centre study revealed that only 38% of graduating students felt confident about prescription writing. In my view, these figures underscore that the transition crisis is not a collection of isolated student anxieties but rather a systemic flaw in institutional design. This editorial explores the mismatch between academic preparation and the practical demands of clinical practice. It focuses on key areas of difficulty, including task prioritisation, responsibilities, escalation of concerns, documentation, and communication under pressure.

Learning during medical school is often heavily based on observation rather than active participation, and opportunities for independent decision-making remain limited. Consequently, students frequently experience insufficient exposure to the workload, administrative demands, and clinical responsibilities of a newly qualified doctor. Together, these factors widen the gap between theoretical knowledge and clinical practice, creating an intensely steep learning curve upon entering the healthcare workplace. Ultimately, this abrupt transition compromises graduate confidence and introduces vulnerabilities into patient safety.

Some strategies to address this gap include early clinical exposure, structured shadowing programmes, simulation-based training, and trust-level induction processes. These interventions can facilitate bridging the divide between theory and clinical practice. This can then improve preparedness and reduce early-career stress.

Strengthening preparedness at the point of transition may improve both clinician well-being and the quality of care delivered to patients. However, achieving this requires coordinated efforts from medical schools, healthcare institutions, educators, and regulatory bodies. Medical schools should provide greater opportunities for supervised responsibility, simulation-based learning, and practical clinical skills training, while hospitals should support graduates through structured induction programmes, shadowing opportunities, and ongoing mentorship. Addressing the gap between undergraduate training and clinical practice should therefore be prioritised not only as an educational concern but also as a patient safety imperative. By working collaboratively to better prepare future doctors for the realities of clinical practice, stakeholders can facilitate a safer transition into the workforce and improve outcomes for both clinicians and patients.

## Editorial

Medical students often leave university with a strong knowledge base about a wide range of conditions and treatments. Throughout medical school, they learn about countless medications, their mechanisms and the pathophysiology of conditions. However, the transition to working as a newly qualified doctor often exposes a gap between knowledge and the application of this knowledge in clinical practice. Despite extensive academic preparation and passing multiple exams, many newly qualified doctors report difficulty adapting to the pace, responsibilities, and decision-making demands required in real-world practice [1].

This editorial explores the mismatch between the undergraduate training commonly provided at medical schools and the challenges faced when starting as a newly qualified doctor.

Key challenges

Task Prioritisation

Newly qualified doctors are required to triage multiple tasks based on importance and urgency, often with limited supervision. Task prioritisation is recognised as a significant challenge for clinicians, particularly during out-of-hours periods when multiple competing demands arise simultaneously [2]. Clinical practice requires rapid prioritisation of tasks for multiple patients. The challenge then lies in delegating tasks to other members of the multidisciplinary team.

The way tasks are presented in a paperless system is also important to consider. In my opinion, the modern shift toward paperless electronic systems has fundamentally altered the cognitive burden on new resident doctors. On a busy ward round or out-of-hours shift, a graduate is no longer just processing raw clinical information; they are forced to act as frontline data triagers. Many clinicians have reported multiple false alarms, which can cause alarm fatigue [3]. Although some alerts may be irrelevant or less important, categorising and prioritising them can cause decision fatigue. Decision fatigue can impact various decisions regarding ordering tests, medication prescribing, and procedures negatively [4]. Even seemingly minor errors in prioritisation may contribute to patient harm [5].

Medical staff understand the importance of task prioritisation in a clinical setting. A cross-sectional study highlighted this gap in training. Therefore, it is essential for medical schools to incorporate this into medical education [6].

Prescribing

Medication and fluid prescribing represent one of the earliest high-stakes responsibilities [7]. The landmark UK EQUIP study highlighted that while Foundation Year 1 (FY1) doctors write the vast majority of hospital prescriptions, they face a staggering 8.4% error rate-nearly double that of senior consultants [8]. Prescribing errors can lead directly to patient harm. Newly qualified doctors are responsible for the majority of prescribing errors in hospitals [9], although it might not have been their sole decision when prescribing. Errors often arise not from lack of knowledge, but from unfamiliarity with using new systems, ongoing departmental pressure and dosing errors [10]. Poor senior support and the fear of appearing incompetent can also cause prescribing errors [11].

Escalation

It is vital for new doctors to escalate concerns to the right person at the right time using the right methods. New doctors may have a lower threshold for escalation to senior colleagues if they are taking care of a patient whose clinical situation is not familiar to them. The quality of the handover received, concerns about contacting senior doctors overnight, and previous experience managing similar situations can influence the doctor's decision regarding escalation [12].

Delayed escalation of care can have significant consequences for patient safety. Failure to involve senior clinicians promptly may result in clinical deterioration, delayed treatment, prolonged hospital stay, and mortality. Newly qualified doctors are often the first clinicians to assess deteriorating patients. Therefore, their ability to recognise and respond to clinical decline is important. Clear escalation plans and documented management strategies have been associated with improved confidence in escalation decisions [13].

Medical schools can better prepare students for when to escalate by offering simulation-based training. Assistantships, structured shadowing programmes, and formal teaching of communication frameworks such as SBAR (Situation, Background, Assessment, and Recommendation) may further enhance preparedness for clinical practice. Importantly, students should be taught that seeking senior input is a marker of professionalism and patient advocacy rather than a sign of incompetence.​​​​​​​

Documentation

With minimal practice of documenting notes during medical school, this can quickly become a challenge for new doctors. Evidence from trainee surveys suggests that formal documentation demands increase sharply after graduation, with many resident doctors reporting that they were not adequately prepared for the documentation workload at the start of practice [14]. It is easy to miss important clinical details during a busy ward round, especially when multiple team members are involved in the patients’ care. Clinical notes are the primary mechanism for information transfer between clinicians in shift-based systems [15]. Incomplete documentation leads to missing information, test duplication, and disrupted continuity of care [16]. Updating electronic health records and medico-legal documentation also places a significant cognitive and time burden on resident doctors, often reducing direct patient-facing time. A study revealed that residents often spend 35% to 40% of their workday navigating electronic health records (EHRs), leaving as little as 15% to 25% of their shift for direct, face-to-face patient-facing time [17].

Medical schools can take initiatives such as short programs [18] to increase students’ skills and confidence in documenting notes. Once again, medical students actively taking part during placements and assistantships and observing notes being documented can also be helpful.​​​​​​​

Communication

Communicating with seniors, nurses, and patients under time pressure is a distinct skill not always fully developed during undergraduate training. Communication can range from breaking bad news to explaining a complicated treatment protocol. It can cause delays in treatment, incorrect treatment being provided, or lead to poorer patient outcomes [19].

Why the gap exists

Observational Learning

Many medical schools rely on sending their students on placements in wards, clinics and surgical departments. This means that their learning relies on passive observation rather than active responsibility. This, in turn, can reduce their readiness for independent decision-making when starting as new doctors.

Insufficient Autonomy

Medical students rarely make unsupervised clinical decisions on placements. Conventional clinical placements often offer limited opportunities for students to participate in healthcare delivery or assume responsibility for patient care, leaving graduates with little experience of clinical accountability prior to qualification [20]. This means that the transition to full accountability is abrupt. Greater opportunities for graduated autonomy may help bridge this gap. Assistantships, structured shadowing programmes, and supervised participation in clinical decision-making can allow students to develop confidence while maintaining patient safety.

Limited Exposure to Responsibility

Final-year placements or assistantships may not consistently replicate the real workload, time pressure, and multitasking demands on the ward. Medical student placements are structured with fixed durations and timetabled hours, which may limit sustained exposure to clinical responsibility and continuity of patient care. Wards and clinics can be dynamic, and the responsibility of a new doctor may change in a very short period of time.

Potential solutions

Early Preparation

A longer period of shadowing in the wards where they intend to start working, and a gradual transition of responsibilities, can help bridge the gap between theoretical knowledge and practice [21]. Spending longer hours, at different times of the day and doing night shifts can help prepare them for the variability of jobs and responsibility. Longer shadowing periods may improve preparedness as a new doctor [22]. Furthermore, formalising "near-peer" mentoring systems, where incoming doctors are paired 1-on-1 with outgoing foundation doctors, can provide realistic, psychological, and practical survival strategies that senior staff might overlook.

Simulation

High-fidelity simulation can expose students to prescribing errors, acute deterioration, and prioritisation under pressure in a safe learning environment [23]. It can test and teach students how to handle such situations in a calm manner and prevent any dangerous mistakes. To maximise efficacy, these simulations should be multi-disciplinary, involving nursing and pharmacy staff. This mirrors real-world ward dynamics, teaches crisis resource management (CRM), and flattens harmful clinical hierarchies that often prevent new doctors from speaking up.

Structured Induction

Robust trust-level inductions focusing on prescribing systems, escalation pathways, and documentation tools improve early confidence and safety. The use of lectures, videos and guides can enable new doctors to catch up quickly. Holding inductions annually can ensure that staff are up to date with the new systems and pathways. Targeted induction programmes for medical students can improve preparedness and confidence among newly qualified doctors [24].

Enhanced Technological and Administrative Support

A significant barrier to transition is cognitive overload from navigating different IT systems and administrative burdens. Implementing standardised Electronic Health Record (EHR) interfaces, paired with easily accessible digital "cheat sheets" or localised mobile applications, can drastically reduce prescribing and administrative errors in the first few weeks.

Institutional Culture and Psychological Safety

Finally, structural interventions must be supported by cultural change. Evidence from healthcare systems research demonstrates that psychological safety is strongly associated with reduced error rates and improved team performance. Formal mechanisms, such as Schwartz Rounds or structured reflective sessions, may help resident doctors process early clinical stressors [25]. In addition, leadership behaviours that actively encourage escalation and normalise uncertainty are essential in mitigating barriers to help-seeking behaviour among new doctors.

Conclusion

The transition from medical student to newly qualified doctor represents a critical juncture where systemic gaps in preparedness may adversely affect both resident doctor wellbeing and patient safety. However, emerging evidence suggests that targeted, multi-faceted interventions can improve early clinical performance and preparedness. Studies evaluating extended shadowing, structured induction programmes, and near-peer mentoring have reported improvements in perceived confidence and reductions in early prescribing errors during the initial postgraduate period [26]. In addition, reflective interventions such as Schwartz Rounds have been associated with improvements in staff wellbeing and reductions in burnout symptoms among healthcare professionals, supporting their role in managing early-career psychological stress [25].

Therefore, addressing this transition gap should be prioritised not only as an educational improvement but as a patient safety-relevant intervention supported by an evolving evidence base. From the author’s perspective, healthcare organisations should consider moving beyond brief, passive induction models towards longitudinal, structured support incorporating simulation-based training, supervised early practice, and formalised reflective spaces. However, implementation should be context-sensitive and aligned with local resources and training structures.

Future research should focus less on repeatedly characterising the transition deficit and more on evaluating the long-term clinical outcomes, cost-effectiveness, and scalability of structured transition interventions across different healthcare systems. Strengthening this transition is likely to improve early-career competence, reduce preventable clinical errors, and enhance the safety and quality of patient care during this high-risk phase of medical training.
